# Blood pressure self-monitoring in pregnancy: examining feasibility in a prospective cohort study

**DOI:** 10.1186/s12884-017-1605-0

**Published:** 2017-12-28

**Authors:** Katherine L. Tucker, Kathryn S. Taylor, Carole Crawford, James A. Hodgkinson, Clare Bankhead, Tricia Carver, Elizabeth Ewers, Margaret Glogowska, Sheila M. Greenfield, Lucy Ingram, Lisa Hinton, Khalid S. Khan, Louise Locock, Lucy Mackillop, Christine McCourt, Alexander M. Pirie, Richard Stevens, Richard J. McManus

**Affiliations:** 1 Nuffield Department of Primary Care Health Sciences, University of Oxford, Oxford, OX2 6GG UK; 2 Institute of Applied Health Research, University of Birmingham, Edgbaston, Birmingham, B15 2TT UK; 3 Obstetrics & Maternal Medicine, Birmingham Women’s Hospital, Edgbaston, Birmingham, B15 2TG UK; 4 Barts and the London School of Medicine and Dentistry, Queen Mary University of London, London, E1 2AD UK; 5 Health Services Research Unit, University of Aberdeen, Aberdeen, UK; 6 Oxford University Hospitals NHS Trust, Women’s Centre, John Radcliffe Hospital, Oxford, OX3 9DU UK; 7 City University London, Northampton Square, London, EC1V 0HB UK

**Keywords:** Hypertension, Self-monitoring, Pregnancy, Pre-eclampsia

## Abstract

**Background:**

Raised blood pressure (BP) affects approximately 10% of pregnancies worldwide, and a high proportion of affected women develop pre-eclampsia. This study aimed to evaluate the feasibility of self-monitoring of BP in pregnancy in women at higher risk of pre-eclampsia.

**Methods:**

This prospective cohort study of self-monitoring BP in pregnancy was carried out in two hospital trusts in Birmingham and Oxford and thirteen primary care practices in Oxfordshire. Eligible women were those defined by the UK National Institute for Health and Care Excellence (NICE) guidelines as at higher risk of pre-eclampsia. A total of 201 participants were recruited between 12 and 16 weeks of pregnancy and were asked to take two BP readings twice daily three times a week through their pregnancy. Primary outcomes were recruitment, retention and persistence of self-monitoring. Study recruitment and retention were analysed with descriptive statistics. Survival analysis was used to evaluate the persistence of self-monitoring and the performance of self-monitoring in the early detection of gestational hypertension, compared to clinic BP monitoring. Secondary outcomes were the mean clinic and self-monitored BP readings and the performance of self-monitoring in the detection of gestational hypertension and pre-eclampsia compared to clinic BP.

**Results:**

Of 201 women recruited, 161 (80%) remained in the study at 36 weeks or to the end of their pregnancy, 162 (81%) provided any home readings suitable for analysis, 148 (74%) continued to self-monitor at 20 weeks and 107 (66%) at 36 weeks. Self-monitored readings were similar in value to contemporaneous matched clinic readings for both systolic and diastolic BP. Of the 23 who developed gestational hypertension or pre-eclampsia and self-monitored, 9 (39%) had a raised home BP prior to a raised clinic BP.

**Conclusions:**

Self-monitoring of BP in pregnancy is feasible and has potential to be useful in the early detection of gestational hypertensive disorders but maintaining self-monitoring throughout pregnancy requires support and probably enhanced training.

**Electronic supplementary material:**

The online version of this article (10.1186/s12884-017-1605-0) contains supplementary material, which is available to authorized users.

## Background

Hypertension in pregnancy, (defined as blood pressure (BP) ≥140/90 mmHg), and pre-eclampsia (where there is proteinuria ≥300 mg/24 h additonally after 20 weeks, gestation) results in substantial maternal morbidity and mortality worldwide [1, 2]. Furthermore, hypertension during pregnancy has been linked to the development of chronic hypertension and an increase in lifetime cardiovascular risk [3]. Raised BP in pregnancy also carries a risk for the baby: pre-eclampsia is associated with fetal growth restriction, low birth weight, preterm delivery, respiratory distress syndrome, and admission to neonatal intensive care [4–6].

Pre-eclampsia causes widespread vascular endothelial dysfunction in the mother that can cause significant increases in BP [7, 8]. Raised BP in the absence of proteinuria is a risk factor for pre-eclampsia. Most cases of pre-eclampsia are asymptomatic in the early stages. Early hypertensive treatment and timely delivery can prevent morbidity and potentially mortality, hence improving detection is important [9].

Inadequate management of raised blood pressure, in particular systolic hypertension, was a key finding requiring action in the 2005–2008 UK Confidential Enquiry into Maternal Deaths [10]. While the maternal death rate from pre-eclampsia and eclampsia has since fallen in the UK, it remains a leading cause of preventable maternal death [9]. The current UK National Institute for Health and Care Excellence (NICE) guideline recommends BP monitoring at routine antenatal visits with increased frequency for those at higher risk of pre-eclampsia, but without defining the testing interval [11]. A large number of women develop pre-eclampsia between antenatal visits, [12] with potentially serious consequences [13]. BP self-monitoring could improve detection of hypertensive disorders [14–16]. However, there is no consensus regarding monitoring protocols or home diagnostic thresholds.

The Blood Pressure Self-Monitoring in Pregnancy (BuMP) study was a prospective cohort study which aimed to evaluate the feasibility and acceptability of a patient controlled intervention to detect hypertension in pregnancy through self-monitoring, in higher risk women. A qualitative study of women’s experiences of self-monitoring will be reported separately.

## Methods

A protocol was developed and registered for a feasibility study of self-monitoring of BP in pregnancy utilising experience from previous trials of self-monitoring in hypertension [17–19]. (National Institute for Health Research (NIHR) Central Research Network (CRN) Portfolio number: 14,151).

### Study participants

Eligible pregnant women were those able and willing to self-monitor BP and defined by the NICE as at higher risk of pre-eclampsia on the basis of any of the following risk factors; aged 40 years or older; nulliparity (first pregnancy); pregnancy interval of more than 10 years; family history of pre-eclampsia; previous history of pre-eclampsia; history of hypertension in pregnancy, body mass index of 30 kg/m^2^ or above at booking; pre-existing vascular disease such as hypertension; pre-existing renal disease; or multiple pregnancy [11]. Potentially eligible women were identified and provided with information about the study at routine antenatal appointments. Recruitment took place in two hospital trusts (in Oxford and Birmingham) and 13 primary care practices across Oxfordshire by midwives, general practitioners and medical staff.

Potential participants attended a study appointment between 12 and 16 weeks of pregnancy, in addition to their usual antenatal appointment, at which informed consent was gained, baseline history and measurements taken, a questionnaire completed regarding lifestyle and demographics and training regarding self-monitoring was delivered. Follow-up occurred at the time of routine antenatal appointments at 16, 28 and 36 weeks of pregnancy. Women requiring admission to hospital or additional clinic BP monitoring were asked to continue to self-monitor if they felt able. A participant flowchart is given in Additional file 1: Figure S1.

### Blood pressure self-monitoring

Participants self-monitored BP using an automated electronic sphygmomanometer validated for use in pregnancy and pre-eclampsia (Microlife WatchBP home) and were asked to self-monitor taking two measurements both morning and evening, following 5 min at rest, three days a week (Monday, Wednesday and Friday) [15, 20]. Participants were provided with a simple colour coded guideline including recommendations to contact relevant health professionals in the case of high or low BP readings (Additional file 2: Figure S2). Little is known about self-monitored BP thresholds in pregnancy. Whilst self-monitoring to monitor essential hypertension typically uses lower thresholds at home compared to clinic, data from population studies suggests that out-of-office measurement is much closer to clinic BP levels in the absence of selection on the basis of raised clinic BP (typical in hypertensive populations) [21, 22]. Furthermore, setting lower home thresholds in this observational study might have led to inappropriate contact with maternity services and anxiety. Therefore, in the guideline given to the women, BP thresholds were set at the same level as clinic thresholds.

Self-monitored BP readings were automatically recorded by the monitors, additionally participating women were asked to note readings in a diary and could optionally text their results via ‘Florence,’ an NHS telemonitoring service (http://www.getflorence.co.uk/) which provided feedback if any action was required. Health care professionals were not blinded to the self-monitored readings.

For analysis, the BP readings downloaded from the monitor were used in preference to diary readings. Minimum criteria for mean self-monitored BP were defined using standard criteria as: at least 12 readings over at least 4 days, plausible levels (systolic BP 70-250 mmHg and diastolic BP 40–150 mmHg), and without an implausible drop between readings (>50% decrease in successive readings) (Additional file 3: Figure S3) [23]. Clinic readings taken as part of usual care were recorded directly from the maternity notes.

### Outcome measures

The primary outcomes were numbers recruited and retained, and persistence of self-monitoring. Retention was defined as the proportion of women recruited who remained in the study until miscarriage or delivery or final follow-up at 36 weeks, whichever was longer. Persistence was defined as the proportion of women who self-monitored until miscarriage or delivery or final follow-up at 36 weeks, whichever was longer.

Secondary outcomes, included the difference between mean self-monitored and clinic BP (systolic and diastolic); and the performance of self-monitored BP in the detection of gestational hypertension compared to the reference of clinic BP.

Final diagnostic verdicts were provided by an obstetric medicine consultant (LM) or an obstetrician (AP), who reviewed the antenatal notes from usual care. These diagnoses were independently reviewed by RM and where more than one diagnosis was recorded, the final diagnosis for that pregnancy was taken (for example gestational hypertension that developed subsequently into pre-eclampsia was defined as the latter). Birthweight centiles of the babies born in the study were calculated using the customised weight centiles GROW (Gestation Related Optimal Weight) [24].

### Sample size calculation

Feasibility studies do not require a formal sample size calculation but a sample of 200 women was considered sufficient to both allow experience with self-monitoring and to include women who developed pregnancy induced hypertension subsequently [25].

### Statistical analysis

#### Persistence

A Kaplan-Meier (KM) survival curve was plotted to describe the persistence of self-monitoring, based on the dates of participants’ final home readings. The plot was constructed on participants from 20 weeks gestation onwards to allow for the fact that women started monitoring at different times between 10 and 18 weeks. Women were censored at delivery or dropout.

#### Mean differences

Self-monitored and clinic BP readings were plotted between weeks 12 and 38 weeks for participants who had both self-monitored and clinic BP data in the same week of pregnancy. Data on clinic BP readings were extracted from the participants’ standard antenatal records. Home BP readings were averaged across the same week as a particular clinic reading.

#### Time to first raised reading

Further KM survival curves were produced from 12 to 40 weeks to present the time to the first raised BP, separately for home and clinic BP readings in women who had both types of readings. Rather than using average BP for home and clinic, this analysis used raised BP readings on a single occasion. A raised clinic BP was defined as a reading recorded in the clinical record ≥140/90 mmHg. The self-monitoring instructions advised women to measure their own BP at least twice per session and to act on the second reading. The first reading was therefore dropped from the base case analysis. Because home readings were repeated if raised, a raised home reading was defined as the final reading of any monitoring “session” ≥140/90 mmHg. Session length was defined as 15 min for high readings (defined as ≥150/100 mmHg) and 5 h for raised readings (140–149/90-99 mmHg), in order to capture data collected in accordance with the participants’ instructions. [Additional file 2: Figure S2]. The survival curves were compared using a log-rank test.

#### Diagnostic accuracy statistics

Women were classed as having hypertension based on home readings if they had at least one episode of raised home BP (Additional file 4: Table S1). Another classification was based on clinic BP readings (Additional file 4: Table S1). These classifications were compared with the final clinical diagnosis of hypertension, the gold standard. Sensitivity, specificity, positive and negative predictive values were produced for both home and clinic BP monitoring.

#### Sensitivity analyses

For time to first raised reading, considered the alternative strategies of including the first home reading of each session, and using a lower threshold for raised home readings of 135/85 mmHg (retaining 140/90 mmHg for clinic).

All analyses were carried out using STATA 12SE (StataCorp, LP).

### Ethical approval

A favourable ethical review for this study was obtained from South Central – Oxford Research Ethics Committee B (reference; 12 SC 0625, 12/12/2012).

## Results

### Participant recruitment

Two hundred one participants were recruited between April 2013 and January 2014, mostly through secondary care (*n* = 162, 81%) with a further 39 (19%) recruited via 13 general practice sites. Women were recruited at between 10 and 18 weeks of pregnancy and followed thereafter. At baseline, they had a mean age of 31.4 (SD 5.5), a BMI of 28.1 (SD 6.6), and a mean clinic BP of 117 ± 10/71 ± 9 mmHg (Table 1). Few women had self-monitored BP previously (4.8%). Participants showed similar levels of anxiety to normative data from working adult women in the age range (STAI: 33.7 [BuMP] vs 35.0 [working adult women aged 24–35]) [26]. Half (50%) reported having professional qualifications or a degree (vs 43% of women aged 25–34 with a degree level qualification or above nationally) [27].

**Table 1:** Baseline characteristics

	Number of participants	Mean(SD) or %
Demographics		
Age (years)	201	31 (6)
BMI^d^	198	28 (7)
Ethnicity		
White	157	78
Mixed	8	4
Asian or Asian British	18	9
Black or Black British	8	4
Chinese or other	6	3
Unknown/not disclosed	4	2
Eligibility^a^		
Age ≥ 40	18	9
BMI ≥30	61	31
Multiple pregnancy	35	17
First pregnancy	87	43
Diagnosed with high BP pre-pregnancy	18	9
High BP in previous pregnancy	42	21
Pre-eclampsia in previous pregnancy	24	12
Family history of pre-eclampsia	39	20
Pregnancy interval > 10 yrs	6	3
Renal disease	6	3
Clinic BP at baseline^b^^,^^d^		
Systolic	200	118 (10)
Diastolic	200	71 (9)
Education (highest)		
Professional qualifications or degree	101	50
School qualifications only	74	37
No formal qualifications	7	4
Unknown	19	9
Other baseline characteristics		
Converted STAI score^c^	179	34 (11)
Measured BP at home previously	9	25

^a^Participants required one or more eligibility criteria

^b^Clinic BP at baseline included readings up to 18 weeks pregnancy

^c^Six-item version of the State-Trait Anxiety Inventory (STAI-6) was converted pro-rata to range 20–80.

^d^There was missing baseline information from one participant and 2 further participants did not wish to provide BMI data

Babies born in the study had a mean GROW birthweight centile of 45.7 (SD 29.2, *n* = 166).

### Retention in the study and persistence with self-monitoring

**Fig. 1:**
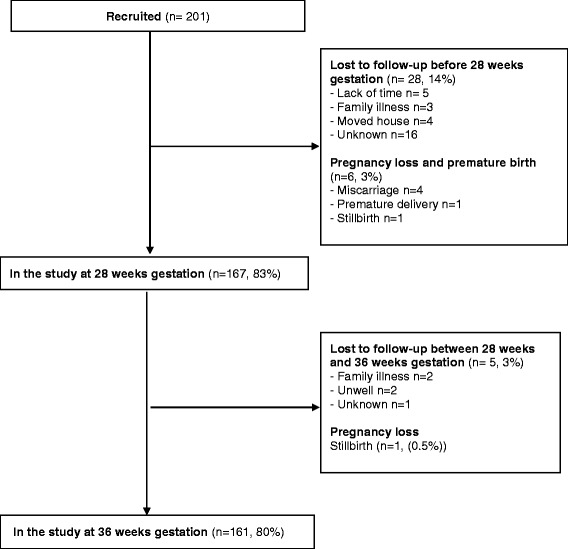
Participant flow through the study. Note: Women attending clinic for follow up did not necessarily provide useable home BP data

Of the 201 women who entered the study, 168 (84%) continued until final follow-up at 36 weeks (or delivery/miscarriage if earlier) of whom 161 (80%) were in the study at 36/40 gestation (Fig. 1). The average gestation at delivery was 38.2 weeks (SD 2.2).

**Fig. 2:**
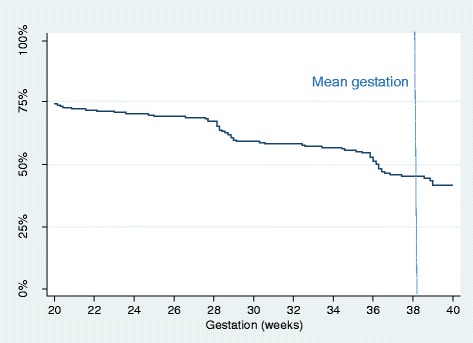
Persistence of self-monitoring. Of 162 who self-monitored, 160 started before 20 weeks gestation and, of these, 12 stopped at or before 20 weeks. The graph presents the remaining 148 patients (74% of the 201 total population, and 91% of the 162 who self-monitored). Mean gestation at delivery is indicated by the dotted line (38.2 weeks)

One hundred sixty-two (81%) women provided any suitable self-monitored BP data for the analysis, a total of 16,940 home readings (Fig. S3) [23]. Of these 162 women, 139 (86%) continued to self-monitor up to 28 weeks or delivery/miscarriage, if sooner, and 107 (66%) continued to 36 weeks or delivery if sooner. Persistence with self-monitoring is shown in Fig. 2.

### Self-monitored and clinic blood pressure readings

**Fig. 3:**
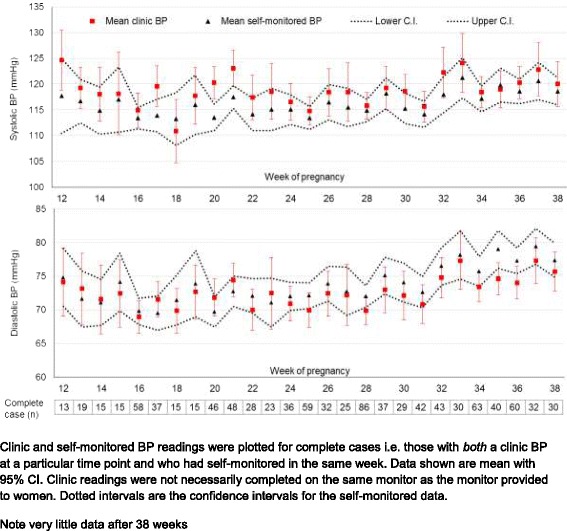
Clinic and home blood pressure readings through pregnancy.

Self-monitored and clinic BP measurements from week 12 to week 38 of pregnancy are shown in Fig. 3. Most systolic and all diastolic point estimates for home and clinic readings were within 5 mmHg and the 95% confidence intervals overlapped for both systolic and diastolic BP other than systolic BP at 20 weeks. However, although this work was not powered to detect such differences, the point estimates for systolic clinic BP tended to be higher than home readings whereas diastolic were if anything reversed.

### Detection of raised blood pressure in women with confirmed gestational hypertension and pre-eclampsia

Final diagnostic verdicts were completed on 157/201 women, including two women who withdrew with permission for notes review (eight sets of notes could not be located or were incomplete and 36 participants withdrew without providing permission for notes review). Overall 28/157 (18%) women developed pregnancy induced hypertension. This can be broken down as follows: considering women developing new hypertension following 20 weeks of pregnancy, 20 (13%) developed gestational hypertension, of whom 5 (3%) went on to develop pre-eclampsia. Of the further 13 (8%) women with chronic hypertension, 5 (3%) developed pre-eclampsia and 3 (2%) had worsening hypertension but not proteinuria.

**Fig. 4:**
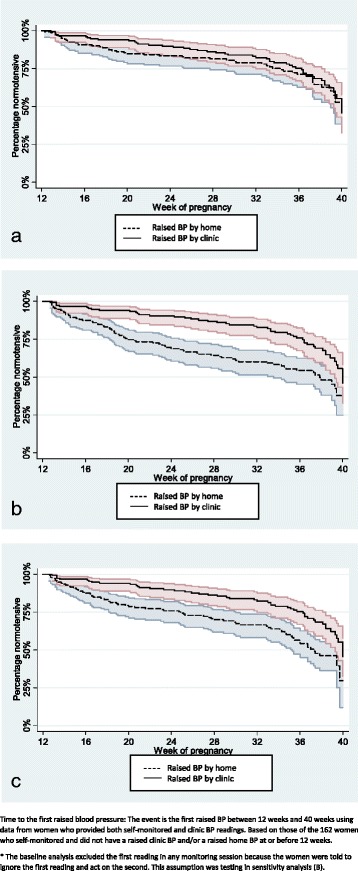
Time to the first raised blood pressure. **a** Time to first raised blood pressure excluding the first home reading taken in any monitoring session and using thresholds for hypertension of 140/90mmHg for home and clinic. n=152*, 50 (33%) had a raised clinic BP reading, 43 (28%) has a raised home BP reading. **b** Sensitivity analysis 1: Time to first raised blood pressure including the first home reading taken in any monitoring session and using thresholds for hypertension of 140/90mmHg for home and clinic. n=151, 49 (32%) has a raised clinic BP reading, 69 (46%) has a raised home BP reading. **c** Sensitivity analysis 2: Time to first raised blood pressure excluding the first reading taken in any monitoring session and using thresholds for hypertension of 135/85mmHg for home and 140/90mmHg for clinic. n=152*, 50 (33%) has a raised clinic BP reading, 69 (45%) had a raised home BP reading

Of the 28 (18%) women with a final diagnosis of pregnancy-induced hypertension, 5 (18%) had stopped self-monitoring by diagnosis. Using a common threshold for raised BP of 140/90 mmHg for both clinic and home BP, of the remaining 23 women, 9 (39%) had a raised home BP prior to raised clinic BP, 5 (22%) had a raised home BP on the same date or after the raised clinic reading, and 9 (39%) only had a raised clinic BP (Additional file 4: Table S1). Considering the 129 (82%) of those with diagnostic verdicts but no final diagnosis of gestational hypertension and/or pre-eclampsia, 25 (19%) had at least one episode of raised home BP and 33 (26%) had at least one episode of raised clinic BP. Of the 152 who did not have a raised clinic BP and/or a raised home BP prior to 12 weeks, the times to detection of first raised BP for clinic and home BPs were not significantly different (Fig. 4a, *p*=0.9).

In detecting hypertension, home monitoring achieved a sensitivity of 61%, specificity of 81%, a positive predictive value of 36% and a negative predictive value of 92% (Additional file 4: Table S2). Additional appointments due to raised home blood pressure were apparently infrequent but this should be treated cautiously as there may have been under reporting.

In sensitivity analyses, two changes in the diagnostic criteria were assessed, including a) the first BP measured at home (Fig. 4b) and b) reducing the self-monitored threshold for raised BP to 135/85 mmHg (retaining 140/90 mmHg for clinic) (Fig. 4c and Additional file 4: Table S1). Both changes increased sensitivity but reduced specificity: both increased the number of women with hypertension initially detected at home from 9 (39%) to 14 (61%) and made the time to detection significantly shorter than detection based on clinic BP readings (*P* < 0.01) (Additional file 1: Tables 2 and 3). However, the inclusion of the first BP measured at each session increased the number of normotensive women with at least one episode of raised home BP from 25 (19%) to 49 (38%). Changing the threshold for raised BP at home to 135/85 mmHg, increased the number of normotensive women with at least one episode of raised home BP from 25 (19%) to 45 (35%).

## Discussion

### Main findings

This study has shown that a range of women with different risk factors for hypertension in pregnancy were willing to take part in a study of self-monitoring of BP. Recruitment took place from a variety of settings, including both primary and secondary care, with the largest numbers of women recruited from hospital sites. Most participants (85%) were successfully trained to self-monitor and, whilst retention in the study was adequate (80%), persistence with self-monitoring to 36 weeks was less so (66%).

In this observational study, clinic readings were used to determine a final diagnosis of hypertension. As expected clinic BP monitoring alone had a sensitivity of 100%, with home monitoring achieving a sensitivity of 61%, specificity was higher with home monitoring (81% vs 74% for clinic monitoring). Home monitoring had a similar positive and negative predictive value to clinic monitoring suggesting that home monitoring is an appropriate intervention to trial formally.

### Is this the correct population to consider for self-monitoring?

This study recruited women at higher risk of pre-eclampsia of which, 18% developed pregnancy-induced hypertension and 6% pre-eclampsia, around double that expected in the overall pregnant population [28]. A population with more than a 1:6 chance of developing a hypertensive disorder of pregnancy would seem appropriate to formally test effectiveness and cost-effectiveness of a self-monitoring intervention. Earlier diagnosis could have a significant impact on pregnancy health care of women, improving the management of hypertension and therefore potentially reducing the number of adverse outcomes relating to gestational hypertension and pre-eclampsia.

### Which are the correct thresholds to use?

Self-monitored readings were similar in value to contemporaneous matched clinic readings for both systolic and diastolic BP in terms of overlapping confidence intervals with point estimates largely within 5 mmHg. Outside of pregnancy, home readings are generally considered to be lower than clinic pressures however this is largely due to selection for a white coat effect inherent in a diagnosis of hypertension on the basis of clinic readings [29]. This study used identical thresholds for action for home and clinic readings, resulting in 39% (albeit of only 23 individuals) detecting definitively raised BP at home prior to the clinic. The sensitivity analysis suggested that earlier diagnosis of hypertension was possible in over 60% with the use of lower home thresholds. This was at the expense of a significant number of false positives. Considering pharmacological intervention does not currently happen until a threshold of 150/100 mmHg in the clinic [28] a regime of increased monitoring at a threshold of 135/85 mmHg at home, with referral to clinic for those with persistently raised home BP of 140/90 mmHg may be an appropriate compromise. Understanding whether this is an appropriate intervention to justify home monitoring on a wide scale to detect pregnancy induced hypertension requires a properly powered trial.

### Strengths and limitations

Considering the availability and uptake of self-monitoring equipment in adults with hypertension, there are remarkably few data concerning its use in pregnancy [30, 31]. This study recruited over 200 participants from diverse settings including inner-city and more rural sites, both teaching and district general hospitals, several GP practices and 20% from minority ethnic groups. The monitor provided to participants was one of the few validated for use in both pregnancy and pre-eclampsia hence the results from self-monitoring should be robust [20]. Almost 20% of women provided no or insufficient self-monitored readings and this may reflect a need for better training in order to maximise retention. 25% of those who were successfully trained to self-monitor did not persist with it, anecdotally for a range of reasons including lack of time, family illness or moving away. Education, training and methods of improving motivation for self-monitoring throughout pregnancy should be considered in future work.

Clinic BP was not systematically measured with identical equipment in each case and so the comparisons with home BP may be flawed. Such between centre differences are inevitable in a pragmatic observational study such as this and reflect the need for stratification by centre in future randomised work.

In this study self-monitoring did not always detect raised readings before clinic readings; this may represent white coat hypertension in these instances and warrants further study [32].

### Comparison with other literature

Self-monitoring of BP will increase women’s involvement with their antenatal care. Previous work has shown that pregnant women are happy to undertake the additional monitoring [33] and find home monitoring more acceptable than more frequent clinic visits [34] or ambulatory monitoring [32, 35]. This was confirmed in our linked qualitative work which will be reported separately [36].

The only randomised trial of self-monitoring of BP in pregnancy to date showed that even a weekly schedule of self-monitoring increased the likelihood of BP monitoring in any given week and therefore increased the likelihood of detection of raised BP [37].

Previous studies that have assessed both clinic and home BP during pregnancy present conflicting results regarding differences in absolute levels, with some finding that home readings were lower on average than clinic readings while others reported the reverse [15, 16, 38–41]. None of these studies used a validated monitor, making comparisons difficult. The largest of these studies found a stable home-clinic difference throughout, similar to that seen here (within 5 mmHg) [40]. Further data regarding relative thresholds for self-monitoring are currently being analysed in an individual patient data meta-analysis [22].

Other work to improve the prediction or early detection of gestational hypertension and pre-eclampsia has focused on risk factors and circulating biomarkers. Biomarkers with promise include factors involved in endothelial and immune response, cardiovascular markers and metabolic factors [42, 43]. The large range of biomarkers under investigation reflects the fact that pre-eclampsia is a multifactorial and multisystem disease [44]. Detection of raised BP by self-monitoring could be complementary to these emerging screening strategies.

## Conclusion

Taken together, these results suggest that women at risk of hypertension in pregnancy are able to self-monitor BP and that this strategy has the potential to detect gestational hypertension prior to clinic readings. However a larger trial, which will require superior training and retention approaches, is required to understand the true effectiveness and cost-effectiveness of self-monitoring of BP in the detection of hypertension in pregnancy.

## Additional files

Additional file 1: Figure S1. Study Procedures. (DOCX 20 kb)

Additional file 2:Figure S2. Participant Interpretation Chart. (DOCX 13 kb)

Additional file 3: Figure S3. Exclusions of home data. (DOCX 21 kb)

Additional file 4: Supplementary Tables. **Table S1. Table S2. Table S3.** (DOCX 15 kb)

## Abbreviations

BP: blood pressure
BuMP study: Blood Pressure Self-Monitoring in Pregnancy study
CRN: Central Research Network
NICE: National Institute for Health and Care Excellence
NIHR: National Institute for Health Research
