# Supplementary material for: Blood pressure self-monitoring in pregnancy: examining feasibility in a prospective cohort study
Source: BMC Pregnancy Childbirth. 2017 Dec 28;17:442. doi: 10.1186/s12884-017-1605-0 (PMC5745883; doi:10.1186/s12884-017-1605-0)
Supplement: Supplementary file 1 — Study Procedures. (DOCX 20 kb) [file 12884_2017_1605_MOESM1_ESM.docx]

**Additional file 1: Figure S1 Study Procedures**

**Self-monitor blood pressure twice a day, three times a week**

**Invitation to participate**

Choice to take part

**Attend 12 or 16 week appointment**

- The study will be explained

- Informed consent

- Initial questionnaire including medical history

- Office blood pressure measurement

- Participant trained to carry out self-monitoring, provided with study information and issued with equipment.

**Week 36: midwife visit**

- Office BP Measurement

- Questionnaire

**Weeks 28: midwife visit**

- Office BP Measurement

- Questionnaire

**Week 6 after delivery**

- Return home monitoring equipment

- Office BP Measurement

- Final questionnaire

- Birth weight recorded
