# Supplementary material for: Blood pressure self-monitoring in pregnancy: examining feasibility in a prospective cohort study
Source: BMC Pregnancy Childbirth. 2017 Dec 28;17:442. doi: 10.1186/s12884-017-1605-0 (PMC5745883; doi:10.1186/s12884-017-1605-0)
Supplement: Supplementary file 2 — Participant Interpretation Chart. (DOCX 13 kb) [file 12884_2017_1605_MOESM2_ESM.docx]

**Additional file 2: Figure S2 Participant Interpretation Chart**

| Level | Blood Pressure | Action |
| --- | --- | --- |
| HIGH | SYS 150 or over  OR  DIA 100 or over | Your blood pressure is high, repeat once more in 5 minutes. If your blood pressure reading is still high you should contact the community midwife, GP surgery or out of hours service as soon as possible (within 4 hours). |
| RAISED | 140-149  OR  90-99 | Repeat the BP measurement after 4 hours.  If it remains raised or you have any symptoms associated with pre-eclampsia (see below) contact a midwife or GP within 12 hours |
| NORMAL | SYS 85-139  OR  DIA 90 or less | Your BP is normal.  This is fine provided that you have no other symptoms  Routine ANC (standard visits) |
| LOW | SYS 85 or less | Your blood pressure is low.  Contact midwife within 24 hours  or within 4 hours if symptomatic |

SYS – systolic, DIA – diastolic

You should seek immediate medical help if you experience any symptoms of pre-eclampsia, which include: severe headache, problems with vision, such as blurred vision or lights flashing before the eyes, severe pain just below the ribs, vomiting, sudden swelling of the face, hands or feet - regardless of your blood pressure readings. If at any time you are concerned about your, or your baby’s, health you should contact your community midwife or seek other medical advice.
