# Supplementary material for: Blood pressure self-monitoring in pregnancy: examining feasibility in a prospective cohort study
Source: BMC Pregnancy Childbirth. 2017 Dec 28;17:442. doi: 10.1186/s12884-017-1605-0 (PMC5745883; doi:10.1186/s12884-017-1605-0)
Supplement: Supplementary file 3 — Exclusions of home data. (DOCX 21 kb) [file 12884_2017_1605_MOESM3_ESM.docx]

**Additional file 3: Figure S3 Exclusions of home data**

* A BP reading is a data record which may include a systolic BP, a diastolic BP or both.

** Based on editing criteria of Stergiou 1998: no home BP readings were excluded for having less than 4 days of home readings. Testing for implausible drops in BP (>50% decrease in successive readings) resulted in the exclusion of 7 systolic BPs and 144 diastolic BPs, but there were no readings where both systolic and diastolic BPs were excluded.

† Time of BP readings was recorded to the nearest minute. BP readings that had the same time were averaged.

‡ Testing for implausible BP readings (systolic BP<70 or >250mmHg and diastolic BP<40 or >150 mmHg) excluded 7 systolic BPs and 54 diastolic BPs, and there was only one reading where both the systolic and diastolic BPs were excluded.

§ Women were asked to measure blood pressure on two occasions, twice daily for three days per week and to act on the second blood pressure reading on each occasion (see supplementary Fig 2)
