# Supplementary material for: Blood pressure self-monitoring in pregnancy: examining feasibility in a prospective cohort study
Source: BMC Pregnancy Childbirth. 2017 Dec 28;17:442. doi: 10.1186/s12884-017-1605-0 (PMC5745883; doi:10.1186/s12884-017-1605-0)
Supplement: Supplementary file 4 — Supplementary Tables. Table S1. Table S2. Table S3. (DOCX 15 kb) [file 12884_2017_1605_MOESM4_ESM.docx]

**Additional file 4: Table S1** Diagnostic criteria

|  | Base case analysis | Sensitivity analysis 1 | Sensitivity analysis 2 |
| --- | --- | --- | --- |
| Included/excluded first home reading | Excluded | Included | Excluded |
| Thresholds for clinic BP | 140/90 mmHg | 140/90 mmHg | 140/90 mmHg |
| Thresholds for home BP | 140/90 mmHg | 140/90 mmHg | 135/90 mmHg |

**Additional file 4: Table S2** Diagnostic accuracy statistics

|  | Home | Sensitivity analysis 1 | Sensitivity analysis 2 | Clinic |
| --- | --- | --- | --- | --- |
| Sensitivity | 61% | 78% | 87% | 100% |
| Specificity | 81% | 62% | 65% | 74% |
| Positive predictive value | 36% | 27% | 31% | 35% |
| Negative predictive value | 92% | 94% | 97% | 100% |

Where the gold standard is final diagnosis and the test is having at least one raised home or clinic BP.

**Additional file 4: Table S3** Detection of raised BP in the 23 women who were self-monitoring and had a final diagnosis of hypertension.

|  | Base case analysis | Sensitivity analysis 1 | Sensitivity analysis 2 |
| --- | --- | --- | --- |
| Raised home BP prior to raised clinic BP, n (%) | 9 (39) | 14 (61) | 14 (61) |
| Raised home BP on same date or after raised clinic reading, n (%) | 5 (22) | 4 (17) | 6 (26) |
| Only had a raised clinic BP, n (%) | 9 (39) | 5 (22) | 3 (13) |
